# Does the choosiness of female crickets change as they age?

**DOI:** 10.1242/jeb.241802

**Published:** 2021-06-11

**Authors:** Edith Julieta Sarmiento-Ponce, Stephen Rogers, Berthold Hedwig

**Affiliations:** Department of Zoology, University of Cambridge, Cambridge CB2 3EJ, UK

**Keywords:** Acoustic communication, Female phonotaxis, Track ball, No-choice experiment, *Gryllus bimaculatus*

## Abstract

For crickets, which approach singing males by phonotaxis, the female choosiness hypothesis postulates that young females should be more selective of male calling song patterns than older individuals. However, there is no information about the behavioural preferences of females over their complete adulthood. We analysed phonotaxis in female *Gryllus bimaculatus* throughout their entire adult lifetime and measured the impact of sound amplitude, carrier frequency and the temporal pattern of test songs on their auditory response. Females of all ages demonstrated their best responses to male calling songs with a pulse period of 34–42 ms, a carrier frequency of 4.5 kHz and a sound pressure level of 75 dB. The response profile to somewhat less optimal song types did vary with age, but not in a manner consistent with a simple loosening of selectiveness in older females. Age, however, had an effect on the overall strength of phonotaxis, as very old females showed an overall diminishing response to all song types. Our data suggest that although there are minor changes in the relative preferences of crickets to individual song elements as they age, the breadth of song patterns to which they will perform phonotaxis remains similar across age groups.

## Abstract (precis)

**Summary:** Exposure of female crickets to variations in song patterns over their lifetime does not reveal a broadening of female preference nor support the hypothesis that female choosiness towards song patterns changes with age.

## INTRODUCTION

Animals make choices over their adult life span that directly affect their reproductive success and ultimately their fitness. One such choice is to find suitable mating partners, as considered by life history theory (Ligout et al., 2012). According to the female choosiness hypothesis (Jennions and Petrie, 1997), young females with a full life expectancy should be highly selective and spend substantial time searching for high-quality males. Conversely, older females with little remaining life risk having no progeny if they are too selective and instead their best strategy may be unselective mating. Secondary sexual displays are a potent source of signals through which mate selections can be made (Andersson, 1994).

A distinction has been made between preference functions, which describe how mating preference changes with systematic variation in display stimulus values, and choosiness itself, which has received contrasting definitions (Reinhold and Schielzeth, 2015). Jennions and Petrie (1997) defined choosiness as ‘the effort an individual is prepared to invest in mate assessment’, independent of preference functions (Neelon et al., 2019). Because of the usefulness of preference functions in defining mate choices, various terms have been used to describe their shape: peak preference is the preferred display trait value; tolerance is the width of the preference function at a particular level of response; strength is the degree to which response falls away from the peak preference as stimuli change away from their peak value (an indication of how sharp or flat the preference function is); and responsiveness is the overall height of the preference function and is often used as a proxy for the general motivation to mate (Jennions and Petrie, 1997; Kilmer et al., 2017; Neelon et al., 2019). By contrast, Reinhold and Schielzeth (2015) define choosiness as a consequence of the shape of preference functions: a choosy individual is one with a narrow preference function in which only very particular stimuli elicit a mating response, whereas non-choosy individuals will respond to a wide range of stimuli; that is, a choosy individual has a narrow tolerance and a high strength.

In crickets (Orthoptera: Gryllidae), males rub their front wings together to produce species-specific songs composed of complex series of sound pulses and silent intervals (Otte, 1992). Female crickets walk towards these songs in a behaviour known as phonotaxis. Phonotaxis is a first step in mate selection; it requires recognising and approaching the source of a conspecific calling song while the female is still distant from the male (Popov and Shuvalov, 1977; Weber and Thorson, 1989; Pollack, 2000), and therefore can require considerable effort on the part of the female in order to reach her potential mate (see choosiness as defined by Jennions and Petrie, 1997). Phonotaxis has been used as a proxy for mate choice, but during courtship, females are exposed to further acoustic (Balakrishnan and Pollack, 1996), tactile and contact-chemical cues (Hissmann, 1990; Adamo and Hoy, 1994; Tregenza and Wedell, 1997; Simmons et al., 2013).

Phonotaxis can be measured in the laboratory using trackball systems that measure the performance of females and record their walking distance and orientation when walking towards a sound source (Weber et al., 1981; Schmitz et al., 1982; Doherty, 1991; Hedwig and Poulet, 2004, 2005; Hedwig, 2017), and thus quantify the attractiveness of different song patterns. Such studies have revealed the tuning of responses to different aspects of song stimuli, with female phonotaxis progressively decreasing the further songs deviate from the preferred species-specific values (Thorson et al., 1982; Doherty, 1985b; Hedwig, 2006; Hennig, 2009; Schneider and Hennig, 2012). A change in female preference function may be expected to change the relative attractiveness of different songs, leading to a change in the width or shape of tuning curves, but age may also have effects on absolute performance or responsiveness. Immature crickets may not have fully developed hearing organs (Popov et al., 1994), or lack a strong motivation to engage in phonotaxis prior to egg maturation (Koch and Hoffmann, 1985). Conversely, senescence is associated with the progressive loss of physiological integrity and declining performance (López-Otín et al., 2013). Therefore, calculating relative phonotactic responses fails to fully describe preference functions, may be misleading, and overrates any orientation response if females are unable or unmotivated to walk long distances (Walikonis et al., 1991; Stout et al., 2010). Only by quantitatively measuring the steering component towards a presented song pattern can the attractiveness of different patterns in different crickets be revealed and separated from the females' overall capacity to act on a pattern.

Analysing phonotactic behaviour has allowed the female choosiness to be tested by comparing the phonotactic responses and mating behaviour of young and old crickets in several species, with varying degrees of support: e.g. studies suggest that older females are less selective when choosing a mate in *Acheta domesticus* (Walikonis et al., 1991; Gray, 1999; Mautz and Sakaluk, 2008; Stout et al., 2010), *Gryllus integer* (Prosser et al., 1997) and *Gryllus lineaticeps* (Atwell and Wagner, 2014). However, no effect of age on mating or song preference was found in studies of *G. pennsylvanicus* (Judge et al., 2010) or *Meloimorpha japonica* (Kuriwada and Kasuya, 2006). In *G. assimilis*, both young and old females oriented towards calling song, but older females had a lower responsiveness (Pacheco et al., 2013). Similarly, data on *Teleogryllus oceanicus* (Tanner et al., 2019) are ambivalent: older females respond with shorter latency to calling song but take longer to reach its source than younger crickets. Although some studies of cricket choosiness concur that age plays a critical role, none have continuously followed phonotaxis in individuals throughout adulthood. Based on the choosiness hypothesis, one may expect that elderly individuals will demonstrate weaker selection for male acoustic signal quality.

We systematically varied three acoustic parameters – sound amplitude, carrier frequency and the pulse period of calling songs – and tested the phonotactic responses of individual females throughout adulthood to determine changes in preference function and choosiness. Although *G. bimaculatus* has been intensively studied, few experiments have addressed the ontogeny of phonotaxis (but see Sergejeva and Popov, 1994, for flying females), and systematic longitudinal studies on phonotactic walking have not been performed. The aim of the present study was to quantitatively measure phonotactic performance across the adult lifetime of female crickets, from their first day of adulthood until advanced senescence, by repeatedly exposing them to a standardised trackball-based testing regime.

## MATERIALS AND METHODS

### Animals

Last-larval instar female *Gryllus bimaculatus* De Geer 1773 were obtained from a breeding colony maintained at the University of Cambridge Department of Zoology for many years, which has been occasionally supplemented by stock from commercial insect suppliers based in the UK. On moulting to the last larval instar, females were housed individually and acoustically isolated from males at 25–28°C with a 12 h:12 h light:dark cycle. The crickets were kept under these conditions for the entire duration of the study and fed a broad diet of muesli supplemented with fish flakes. Water was provided in wetted cotton wool pads. Phonotaxis was tested repeatedly in a soundproof chamber (155 cm wide, 75 cm high and 100 cm deep) throughout the adulthood of every female across 45 days. On each day, the crickets were tested with an attractive song pattern that closely resembles natural male calling song [75 dB sound pressure level (SPL), 4.5 kHz and 34 ms pulse period (PP)], and with one other test pattern (see below). Because females were not tested with every song pattern, every day, responses were assigned to one of five age groups for analysis purposes: very young, 1–5 days; young, 6–12 days; mature, 13–24 days; old, 25–36 days; and very old, 37–45 days. For individuals tested more than once with a stimulus type within an age group, mean values were used. Because mating status strongly influences phonotactic responsiveness (Judge et al., 2010; Tanner et al., 2019), we only used isolated virgin females.

### Trackball system

An open-loop trackball system was used to measure phonotaxis (see Hedwig and Poulet, 2004, 2005). Tests were performed in the dark at 25–28°C. Females walked on a rough-textured trackball, 5.6 cm in diameter and weighing 5.3 g (Rohacell 31 IG-F, Evonik, Darmstadt, Germany) (Sarmiento-Ponce et al., 2018).

### Sound stimuli and acoustic parameters

Computer-generated acoustic signals were presented through two speakers (Neo 13S, Sinus Live, Conrad Electronics, Hirschau, Germany) positioned 57 cm in front of the cricket at 0 deg elevation and 45 deg either side of its long axis (Hedwig and Poulet, 2004, 2005). Three acoustic test patterns were played with systematically altered sound amplitude, frequency or pulse period, designed to span the parameters of male calling song, which has a narrow carrier frequency range centred on 4.7 kHz and is composed of discrete 120 ms chirps, with a 240 ms inter-chirp interval, and composed of four pulses at a pulse rate of 25–30 Hz (Ferreira and Ferguson, 2002). Test patterns were played for 30 s first from the left and then right speaker, with 10 s silence before swapping sides. The sound amplitude test consisted of six pulse chirps at 4.5 kHz and 40 ms PP that increased from 40 to 85 dB SPL in 5 dB steps, based on the pattern of Thorson et al. (1982). The root mean square (RMS) of the sound amplitude was measured and calibrated at the position of the cricket (amplifier type 2610, microphone type 4191; Brüel and Kjær, Nærum, Denmark). For the second test, the carrier frequency increased from 2 to 17 kHz while four-pulse chirps with a 40 ms PP were played at 75 dB SPL. For the song pattern test, we followed an established paradigm where the PP increased from 10 to 98 ms in 8 ms steps (Thorson et al., 1982), while keeping the frequency at 4.5 kHz and amplitude at 75 dB SPL. The stimulus order was kept the same throughout all experiments, rather than being randomised, so that results obtained on different days were strictly comparable, even though this could have potentially introduced motivational effects on walking that persist to subsequent stimuli. Such effects are generally minor (Doherty, 1985a), and to adequately control for presentation sequence would have required considerably expanded sample sizes in order to resolve any effects. Experiments were performed from 10:00 to 18:00 h randomly to avoid possible circadian bias.

### Data analysis

For each stimulus, we calculated the total distance walked towards the active speaker (lateral deviation; cm per 60 s), which indicates the strength of phonotaxis (Hedwig and Poulet, 2004, 2005). Statistical analyses were performed with RStudio Team (RStudio, Boston, MA, USA) and NCSS 10 (NCSS, LLC, Kaysville, UT, USA). Details of statistical tests used are given where they appear in the Results. We determined the sound amplitude at which females started performing phonotaxis, using the response to the 40 dB SPL stimulus as a reference, which is subthreshold for the auditory pathway (Nocke, 1972). As the threshold for phonotaxis, we identified the sound amplitude at which the response became significantly different (*P*<0.05) from the 70% quartile of the range of movement during the 40 dB reference stimulus for females of each age cohort using the Wilcoxon signed rank test.

## RESULTS

When presenting our data, we first describe the behavioural response of a representative female and then results from all 20 crickets.

### Effect of sound amplitude

**Fig. 1.**
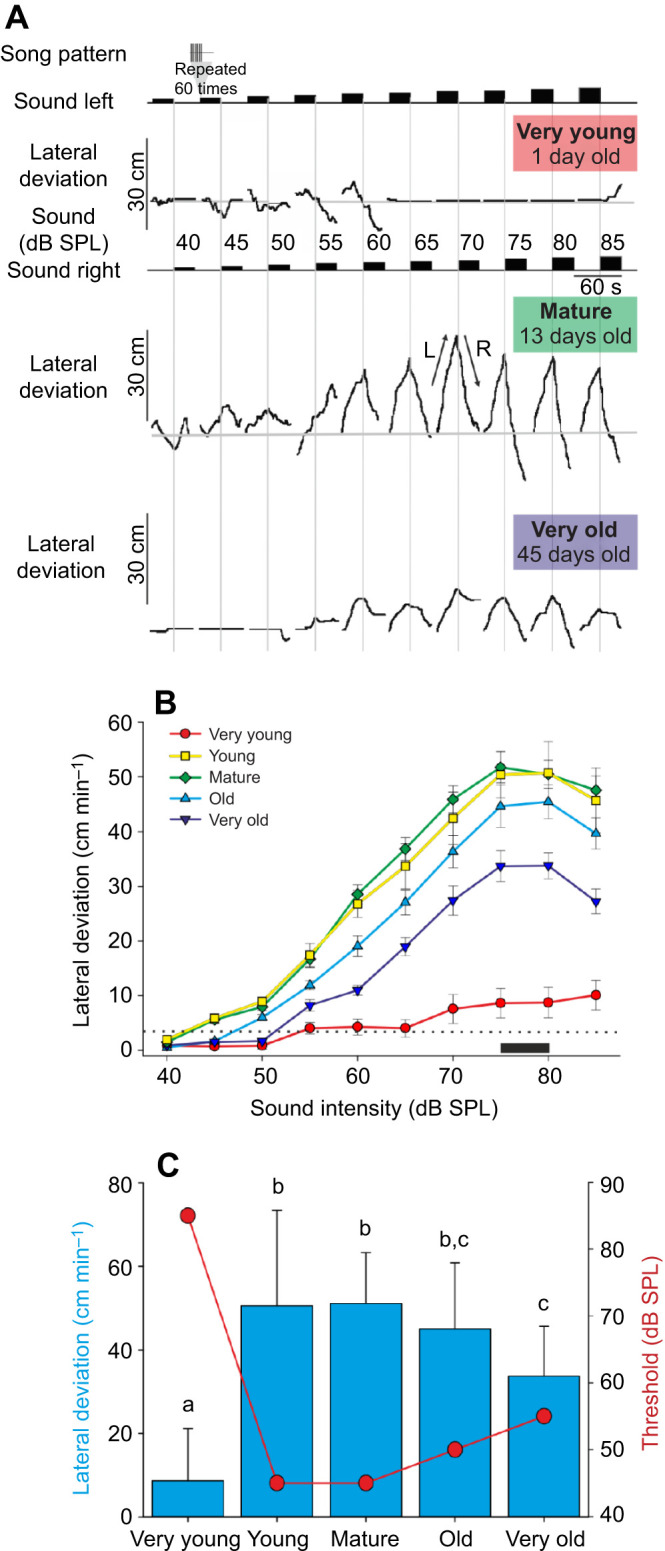
**Phonotaxis to songs of increasing sound amplitude in *Gryllus bimaculatus*.** (A) Response of a female at three different ages. Black rectangles represent 30 s of calling song of escalating amplitude. Vertical grey lines mark a switch in the active speaker. Lateral deviation shows the walking response. L and R indicate walking to the left or right, respectively. (B) Effect of sound amplitude on phonotaxis over the adulthood of 20 female crickets within five age categories. Data are means±s.e.m. (C) Bar chart of mean±s.d. maximum phonotactic responses that occur at 75 and 80 dB sound pressure level (SPL), indicated by black horizontal bar in B. A difference in the letters above the bars indicates that groups were significantly different (*P*<0.05) from each other in Bonferroni-corrected *post hoc* paired comparison tests. The red circles and line indicate the threshold at which walking activity is above the 75th quartile of the range of movement seen in crickets stimulated by sub-auditory threshold calling song at 40 dB SPL (dotted line in B).

On day 1 of adulthood, a very young female walked while the test pattern was played at 40 to 60 dB SPL (Fig. 1A). Her lack of a consistent lateral deviation indicates that walking was not directed towards the sound signal, e.g. at 55 and 60 dB SPL she turned right whilst the song was still coming from the left (Fig. 1A, top). She remained mostly stationary to 65–85 dB SPL song. By day 6, as a young female, she performed phonotaxis to 55 dB SPL (15.3 cm min^−1^), and showed a maximum response to 85 dB SPL (28.6 cm min^−1^, data not shown). As a mature female (day 13), she reliably oriented to the song (Fig. 1A middle), with a threshold of 45 dB SPL (10.0 cm min^−1^). Phonotaxis was strong from 60 to 85 dB SPL, with a maximum lateral deviation of 63.3 cm min^−1^ at 75 dB SPL. The response then decreased at 80 dB SPL to 61.2 cm min^−1^ and further at 85 dB SPL to 56.8 cm min^−1^. Finally, at 45 days (very old), her threshold for phonotaxis was 60 dB SPL (14.8 cm min^−1^); the strongest response occurred at 80 dB SPL (26.3 cm min^−1^), while to 85 dB SPL the response decreased to 12.9 cm min^−1^ (Fig. 1A, bottom). In summary, both her threshold for phonotaxis and her maximum response changed markedly as she progressed though adult life.

Responsiveness (the overall distance walked) across the entire sample of 20 crickets also increased from little activity when very young to a maximum when mature, before gradually declining with age (Fig. 1B). Generally, for all age groups, phonotaxis increased from 45 to 70 dB SPL, with a maximum at 75–80 dB SPL, followed by a slight decrease at 85 dB SPL. The maximum response of very young females, however, occurred at 85 dB SPL, albeit with a very low activity of 10.1±2.8 cm min^−1^. Young and mature adults showed a peak response at 75 dB SPL of 50.7±5.9 and 51.8±2.9 cm min^−1^, respectively. The maximum response of old and very old adults was at 80 dB SPL with 45.5±3.2 and 33.8±2.4 cm min^−1^, respectively (Fig. 1B). We averaged data from 75 and 80 dB SPL to compare maximum responses between age groups. The mean response of very young females (8.7±12.4 cm min^−1^) was 83% less than young females (50.6±22.8 cm min^−1^), which showed the greatest responsiveness. With age, phonotaxis decreased: from 45±15.8 cm min^−1^ in old females (12% less than best response) to 33.7±11.9 cm min^−1^ in very old females (33% less than best response) [repeated-measures mixed model (RM-MM), *F*_4,4_=96.92, *P*=3×10^−4^; Fig. 1C].

The threshold of phonotaxis was 85 dB SPL (10.1±2.8 cm min^−1^) for very young females. For the young and mature age groups, the threshold was 45 dB SPL; in old females, the threshold increased to 50 dB SPL, and it increased further to 55 dB SPL for very old females (Fig. 1C, red circles).

### Effect of song frequency

**Fig. 2.**
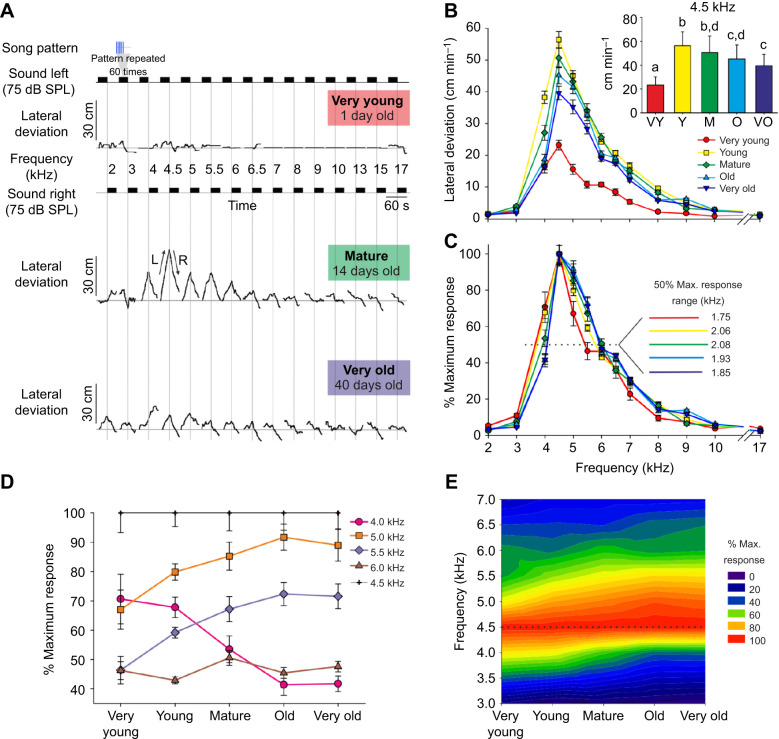
**Phonotaxis varies with both song carrier frequency and age in *G. bimaculatus*.** (A) Response of a female at three different ages to different carrier frequencies of the calling song. Black rectangles represent 30 s of the calling song. Songs were played at increasing sound frequency; changes of speaker are indicated by vertical grey lines. (B) Phonotactic responses of 20 female crickets divided into five age categories (means±s.e.m.). Inset shows mean±s.d. of the maximum phonotactic responses that occur at 4.5 kHz. A difference in the letters above the bars indicates that groups were significantly different (*P*<0.05) from each other in Bonferroni-corrected *post hoc* paired comparison tests. (C) Mean±s.e.m. phonotactic responses at different calling song frequencies expressed as a percentage of the response to 4.5 kHz, for the five age classes. The inset shows the tolerances for the different age classes at 50% of maximum response (dotted line). (D) Change in responsiveness with age for calling song frequencies from 4 to 6 kHz relative to maximum phonotactic response at 4.5 kHz (means±s.e.m.). (E) Contour plot of the strength of the phonotactic response relative to 4.5 kHz, compared by calling song frequency and age class.

On day 1 of adulthood, a very young female walked weakly while the test pattern was presented from 2 to 6.5 kHz and 17 kHz, but she did not orient towards the sound (Fig. 2A, top); she remained stationary between 7 and 15 kHz. As a young female (7 days, data not shown), she performed phonotaxis and her maximum response was to 4 kHz (22.3 cm min^−1^); she did not respond between 6.5 to 17 kHz. When mature (14 days), she responded strongly to 4 kHz (52.4 cm min^−1^) and maximally to 4.5 kHz (72.9 cm min^−1^) (Fig. 2A, middle). There was no phonotaxis from 8 to 17 kHz. Finally, when very old (40 days old), her overall response decreased: the maximum was at 4.5 kHz (27.9 cm min^−1^), while there was no phonotaxis from 7 to 17 kHz (Fig. 2A, bottom).

The response of this female was reflected in the sample of 20 individuals (Fig. 2B). As with song amplitude, phonotaxis increased from little activity in the very youngest crickets, reached a maximum in the young age group, before gradually declining with age. Phonotaxis was confined to a narrow band between 4 and 8 kHz. The preferred frequency was 4.5 kHz for all age groups, with a gradual decrease in response occurring between 5 to 8 kHz (Fig. 2B). Very young females showed the lowest maximum response with 23.2±1.6 cm min^−1^. Substantially stronger phonotaxis occurred when young (56.4±2.6 cm min^−1^) and mature (50.7±3.1 cm min^−1^). The maximum response decreased in the old (45.2±2.6 cm min^−1^) and very old stages (39.6±2.2 cm min^−1^). Young females performed significantly better than when very young or very old (RM-MM, *F*_4,4_=63.53, *P*=7×10^−4^; Fig. 2B inset). At 4.5 kHz, the response when very young was 59% weaker than that of young crickets, while the response of old and very old crickets was 20% and 30% weaker, respectively.

Rescaling the data relative to the maximum response at 4.5 kHz revealed progressive changes in relative frequency preference that occurred with age (Fig. 2C,D). At 4 kHz the response of very young crickets was 70.6±8.4% as strong as their response to 4.5 kHz. As the cohort aged it became progressively less responsive to 4 kHz, until finally the response of very old crickets was only 41.8±8.9% as strong as the response to 4.5 kHz [RM-MM, *F*_4,76_=8.36, *P*=1.2×10^−5^; Tukey–Kramer *post hoc* (T-K) test indicated that very young and young groups were different from old and very old groups; Fig. 2D]. The responsiveness of very young crickets to 5 kHz songs was similar to that to 4 kHz songs (67±6.7% of maximum response), but as they aged the crickets became progressively more responsive to 5 kHz song, so that the response of old and very old crickets was 91.7±4.5 and 89±5.5% as strong, respectively, as their response to 4.5 kHz song (RM-MM, *F*_4,76_=4.34, *P*=0.003; T-K test indicated that the responses of very young crickets were lower than those of old and very old crickets; Fig. 2D). The responsiveness of old and very old crickets to 5 kHz was still significantly lower than their response to 4.5 kHz (paired *t*-test, *t*_19_=−3.03, *P*=0.007 for old and *t*_19_=5.93, *P*=1×10^−5^ for very old crickets). The responses of crickets to 5.5 kHz songs was approximately 20% lower than the response to 5 kHz across all age classes, and the same pattern of rising responsiveness with age was apparent (RM-MM, *F*_4,76_=9.57, *P*=3×10^−6^; T-K test indicated that the responses of very young crickets were different from those of mature, old and very old crickets; Fig. 2D). Finally, by 6 kHz, the responses were between 43 and 50% of the maximum across all ages with no clear pattern discernable (RM-MM, *F*_4, 76_=1.62, *P*=0.177; Fig. 2D). These data suggest that there was a slight increase in preferred calling song frequency as crickets aged of approximately 0.25 kHz and possibly a shift from a sharp preference for 4.5 kHz in very young crickets to nearly equal preference for 4.5 and 5 kHz songs in old and very old crickets (Fig. 2E), although we cannot exclude the possibility that a peak responsiveness occurred between 4.5 and 5 kHz. A transect taken through 50% of the maximum response (dotted line Fig. 2C, inset) does not suggest a consistent broadening of tolerance as crickets age.

### Effect of song pattern

**Fig. 3.**
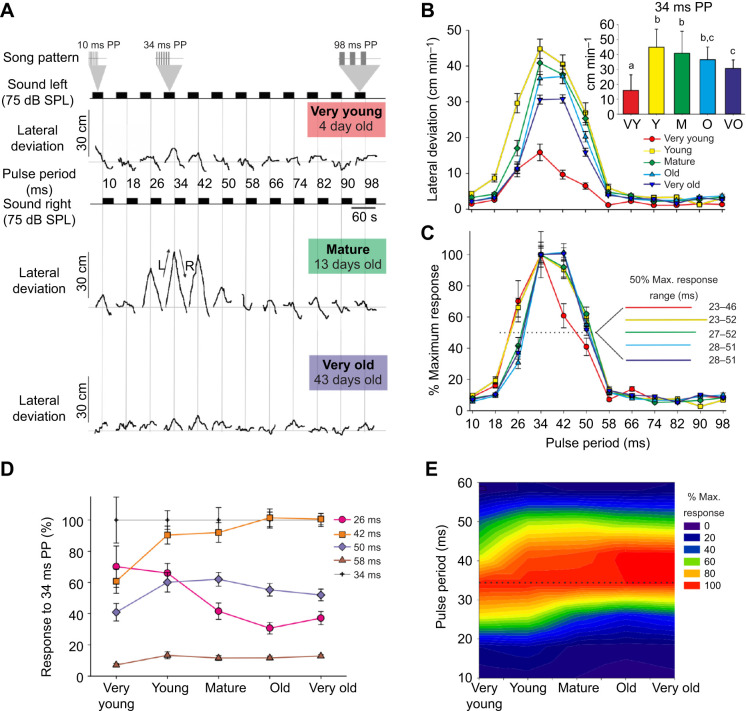
**Song pattern and age affect the extent of phonotaxis in *G. bimaculatus*.** (A) Phonotactic response of a female at three different ages to calling songs with increasing pulse periods (PP; 50% duty cycle). Each new song increased the pulse period by 8 ms (for further details, see Fig. 1). (B) Phonotactic responses of 20 females divided into five age categories to different pulse periods (means±s.e.m.). Inset shows response to songs with 34 ms PP; different letters above bars indicate a significant difference in a Bonferroni-corrected *post hoc* test. (C) Responses of female age groups to different pulse periods expressed as a percentage of the response to song with 34 ms PP. Inset shows the tolerance of the different age groups at 50% of maximum response (dotted line). (D) Change in responsiveness with age for songs with pulse periods from 26 to 58 ms relative to the response to 34 ms PP song (means±s.e.m.). (E) Contour plot of the relative strength of phonotaxis relative to 34 ms PP song, compared by pulse period and age class.

A very young female (4 days) walked without orienting to the sound stimuli from 10 to 26 ms PP. She performed weak phonotaxis to 34 ms PP at 20.9 cm min^−1^, and to 42 ms PP at 14.0 cm min^−1^. From 50 to 98 ms PP, she no longer oriented towards the sound (Fig. 3A, top). When mature (13 days), she oriented weakly towards 10 ms PP, walking at 10.5 cm min^−1^, but she did not respond to 18 ms PP. She had a strong response to 26 ms PP walking at 49.1 cm min^−1^, increasing to a maximum response of 74.7 cm min^−1^ to 34 ms PP, but by 50 ms PP the response was smaller again, at 24.0 cm min^−1^ (Fig. 3A, middle). Finally, when very old (43 days), her phonotaxis was diminished: she oriented to 34 ms PP at 11.1 cm min^−1^, her maximum response occurred to 42 ms PP at 16.3 cm min^−1^, and to 50 ms PP it had decreased to 8.8 cm min^−1^ (Fig. 3A, bottom).

Across the larger sample, females started performing substantial phonotaxis (>10 cm min^−1^) to 26 ms PP; however, its extent depended on their age (Fig. 3B). As with the other song parameters, responses were weakest in the very young age group, rose sharply to a maximum in the young age group before gradually declining with each successive age cohort. The strongest phonotaxis in most ages occurred in response to 34 ms PP (Fig. 3B inset). Very young females had the weakest response (15.8±2.3 cm min^−1^). The largest response (44.8±2.7 cm min^−1^) occurred when females were young, followed by the mature (40.9±3.3 cm min^−1^), old (36.6±1.9 cm min^−1^) and very old (30.6±1.3 cm min^−1^; repeated-measures ANOVA, *F*_4,76_=28.26, *P*=2.2×10^−14^). When very young, crickets walked significantly further towards 34 ms PP than to 42 ms PP (paired *t*-test, *t*_19_=5.38, *P*=4×10^−5^), with an average difference of 39%. The relative difference between the two patterns decreased to just 10% in young crickets (*t*_19_=10.33, *P*=3.1×10^−9^). In mature crickets, the relative difference decreased yet further to 8% (*t*_19_=2.96, *P*=0.008). In old and very old crickets, however, there was no longer any preference for 34 ms PP over 42 ms PP (*t*_19_=−0.61, *P*=0.546 for old crickets, and *t*_19_=−0.507, *P*=0.618 for very old crickets), and indeed the average response to 42 ms PP was slightly higher than to 34 ms PP, but only by 0.5–1%. The longest PP that all ages responded to was 50 ms PP, with again the weakest response given by very young adults (6.5±0.9 cm min^−1^), and the strongest by young adults (27.0±2.8 cm min^−1^; Fig. 3B).

The responses of very young crickets to 26 ms PP were 70.1±13.2% as great as their responses to 34 ms PP, but as crickets aged, their relative responsiveness to this PP declined (RM-MM, *F*_4,76_=6.33, *P*=1.8×10^−4^; T-K test indicated that very young crickets responded more than the mature, old and very old groups, whereas young crickets responded more than old and very old crickets; Fig. 3C,D). Conversely, the response to 42 ms PP relative to 34 ms PP increased as crickets aged (RM-MM, *F*_4,76_=9.15, *P*=4×10^−6^; T-K test indicated that responses of very young crickets were lower than all others; Fig. 3C,D). There was much less difference in relative responsiveness to 50 ms PP as the crickets aged, which ranged from 40 to 60% of the response to 34 ms PP (RM-MM, *F*_4,76_=3.94, *P*=0.006; T-K test indicated that very young crickets were different from young and mature groups, but not from older crickets; Fig. 3D). The response of crickets to 58 ms PP song was substantially less than their maximum response (average range 7.1−13.2%), but the response of very young crickets was still less than that of older crickets (RM-MM, *F*_4,76_=3.11, *P*=0.02; T-K test indicated that very young crickets responded less than young and very old crickets; Fig. 3D).

As crickets aged, there was a slight shift towards preferring slightly longer pulse periods, and the peak of the response became less sharply defined (Fig. 3E), i.e. the strength of the preference function decreased somewhat: old and very old crickets made no distinction between 34 and 42 ms PP. The apparently increased discrimination of very young crickets has to be offset against their very low levels of activity overall. The tolerance of pulse widths at 50% of maximum responsiveness was similar across all ages (Fig. 3C, inset), spanning 23 ms; only young crickets had a somewhat expanded 29 ms range.

### Phonotactic responses over the adult lifetime of the females

**Fig. 4.**
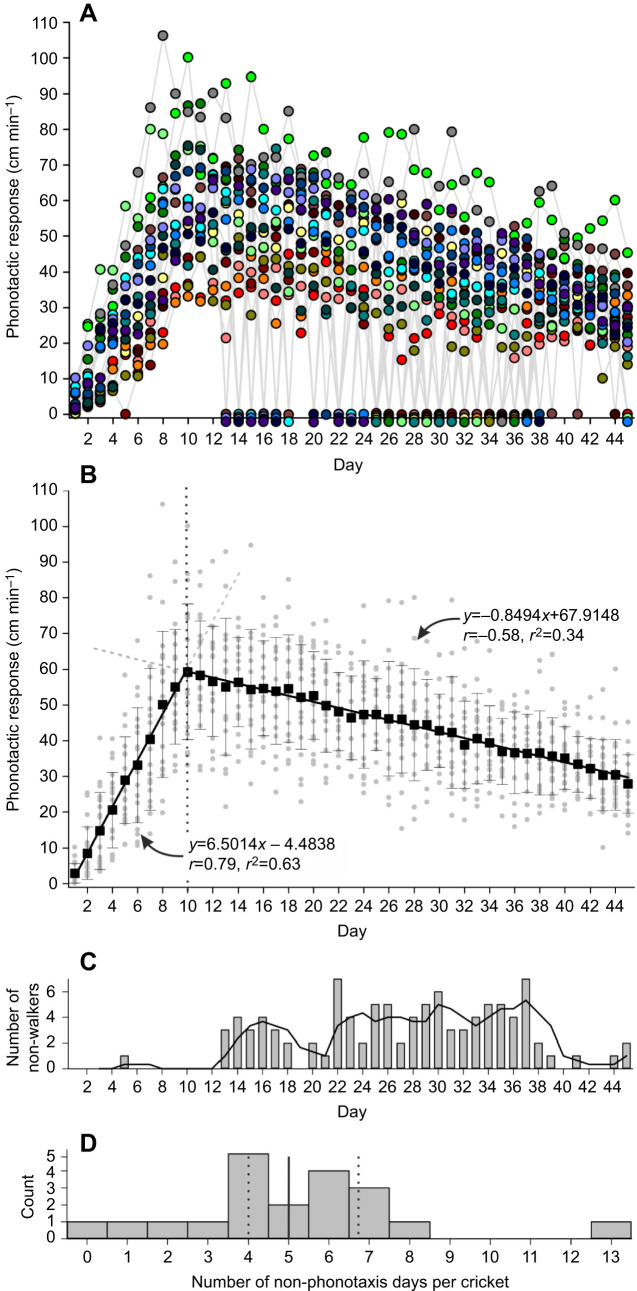
**Phonotactic response over cricket lifetime.** (A) Daily phonotactic responses over the adulthood of 20 females to an attractive calling song (75 dB SPL, 4.5 kHz, 34 ms PP); each individual is represented by a different colour. (B) Scatter plot (excluding non-responses); black squares show means±s.d. The data can be described by two linear relationships spanning from days 1 to 10 and from days 10 to 45. (C) Numbers of females that did not perform phonotaxis each day of the experiment; superimposed is a 3 day running average. (D) Distribution of the number of days that individual crickets did not perform phonotaxis; solid line represents the median and dotted lines the interquartile range.

Every female was tested three times daily with an attractive calling song (75 dB SPL, 4.5 kHz and 34 ms PP); Fig. 4A shows the best phonotactic score out of these three daily tests for each cricket.

The best phonotactic response over time can be represented by two linear relationships, the first spanning from days 1 to 10, and the second from days 10 through to 45 (Fig. 4B), which excludes individuals that failed to walk during any of the three tests (shown as a daily count in Fig. 4C). The first of these relationships shows the rapid increase in phonotaxis as crickets mature on reaching adulthood, with distance moved towards the speaker increasing by 6.5±0.4 cm min^−1^ each day (*y*=6.5*x*−4.5, *r*=0.79, *r*^2^=0.63; RM-MM, *F*_9,170_=101.5, *P*<1×10^−9^). The best phonotactic response occurred on day 10; afterwards there was an equally steady but shallower linear decrease in responsiveness with age, with crickets showing 0.85±0.05 cm min^−1^ less phonotaxis each day (*y*=−0.85*x*+67.9, *r*=−0.58, *r*^2^=0.34; RM-MM, *F*_35,563.1_=27.4, *P*<1×10^−9^). The profiles of each cricket (Fig. 4A) reveal some small variation in the day of transition from rapidly rising response to slow decline.

### Individual variation

The proportion of crickets that did not walk during at least one daily test increased with age (Fig. 4C). During the first 12 days there was only one cricket out of 20 that did not show phonotaxis, and only for 1 day (0.4% of observations). From days 13 to 24, there were 35 occasions where crickets did not perform phonotaxis (median 3 individuals per day, range 0–7, 14.6% of total observations; Fisher's exact test, *P*<1×10^−5^, comparing days 1–12 with days 13–24). From days 25 to 36, there were 51 occasions where crickets did not perform phonotaxis (median 4.5 individuals per day, range 2–6, 21.3% of total observations; Fisher's exact test, *P*=0.0738_,_ comparing days 13–24 with days 25–36). Very old animals, from days 37 to 45, however, were more likely to perform phonotaxis (median 1 animal per day did not perform phonotaxis, range 0–7 crickets daily, 7.8% of total observations; comparison days 25–36, Fisher's exact test, *P*=1×10^−4^).

Only one cricket performed phonotaxis every day. The median number of days that crickets did not perform phonotaxis was five (11.1% of total observations), with an interquartile range of 4–6.75 days (Fig. 4D). No cricket failed to walk for more than 3 days in a row.

## DISCUSSION

We analyzed the phonotaxis of virgin female *G. bimaculatus* over their entire adulthood using a highly sensitive trackball, which gives a quantitative readout of the behavioural performance (Hedwig and Poulet, 2004, 2005). The individuals were repeatedly tested regarding behavioural threshold, carrier frequency and sound pattern across five age groups, which extends previous categorisations (Atwell and Wagner, 2014).

Female preference functions for different song characteristics were mostly stable across all age groups, apart from their responsiveness, which increased approximately three-fold from when crickets were very young compared with the young age group, and then underwent a more gradual decline with ageing so that the responsiveness of the oldest crickets was approximately half that of young crickets. We found some evidence for a minor shift in peak preference with respect to song carrier frequency and pulse pattern with concomitant shifts of the entire preference function, but the tolerance and strength aspects of the preference function remained similar across all age groups. From these observations it must be concluded that there is little change in female choosiness as defined by Reinhold and Schielzeth (2015) during aging. Because long-distance phonotaxis represents a considerable effort to engage in reproduction, it could be argued that choosiness as defined by Jennions and Petrie (1997) declines with age, but this probably represents a decrease in the capacity to walk long distances owing to senescence. Slower and shorter phonotaxis will only reduce mating opportunities, opposite to the effect that decreased choosiness is meant to facilitate.

Repeated testing may be confounded by experience and/or habituation effects. Phonotaxis, however, increased over the first 10 days before gradually declining, and crickets in natural habitats will also be repeatedly exposed to calling songs. We believe that our data therefore provide a robust measurement of phonotaxis throughout adulthood. Mating status influences phonotactic behaviour in different cricket species (e.g. Prosser et al., 1997; Mautz and Sakaluk, 2008; Judge et al., 2010; Tanner et al., 2019). After mating, *G. bimaculatus* females cease performing phonotaxis for at least 16 days (Loher et al., 1993); we therefore only used isolated virgin females.

### Variability of responses

Female crickets show considerable daily variability in phonotaxis (Thorson et al., 1982; Prosser et al., 1997; Pacheco et al., 2013), illustrating the impact of motivational states that cannot be experimentally controlled. We mitigated this effect by testing each animal three times daily and only using its best score. We performed no-choice tests, as exposure to attractive songs affects responses to less-attractive signals (Poulet and Hedwig, 2005), complicating the interpretation of two-choice experiments. Doherty (1985a) reported that the attractive range of PP in choice experiments was similar to that in no-choice experiments but presented anecdotal evidence that some females showed tighter preferences when presented with a choice.

### Onset and development of phonotaxis

Female *G. bimaculatus* produced a low level of phonotaxis within 2 days of reaching adulthood. Sergeyeva and Popov (1990) reported incidences of positive phonotaxis in approximately one-third of tethered flying *G. bimaculatus* after just 1 day of adulthood; Loher et al. (1992) reported the onset of phonotaxis in this species at 5–6 days. Female *Acheta domesticus* showed phonotaxis after 3–5 days (Sakaluk, 1982; Walikonis et al., 1991; Prosser et al., 1997), while in *Gryllus assimilis*, 1- to 7-day-old females did not orient toward male song (Pacheco et al., 2013). We found that the strongest phonotaxis in *G. bimaculatus* occurred on the 10th day of adulthood. Similarly, phonotaxis peaked in *G. assimilis* after 10–13 days (Pacheco et al., 2013) and in *G. lineaticeps*, phonotaxis was stronger at 13 days than at 25 days (Atwell and Wagner, 2014). These data suggest an overall similar development of phonotaxis in different cricket species. We found that phonotaxis increased steeply in a linear fashion over the first 10 days of adulthood followed by a slower linear decrease, suggesting that in *G. bimaculatus*, the development of phonotaxis may be controlled by simple underlying physiological processes. There is a correlation with the timing of egg production (Koch and Hoffmann, 1985), which starts 6–8 days post eclosion, reaches a broad maximum from days 10 to 20 and is followed by a gradual decline towards day 30. Thus, the period of best phonotaxis coincides with maximum egg production, while both egg production and phonotaxis decline in parallel in older females (25–45 days). Our crickets showed pronounced phonotaxis even before egg production and performance improved throughout the maturation process. Female crickets store sperm prior to fertilisation (Simmons, 1986; Loher et al., 1993), and early mating is not necessarily wasted. Furthermore, female *G. campestris* that share a burrow with males experience reduced predation risk (Rodríguez-Muñoz et al., 2011), meaning that phonotaxis could be beneficial even before reproductive maturity.

### Effect of sound amplitude

Very young females significantly responded only to 85 dB SPL songs. The phonotactic threshold substantially decreased in young and mature crickets to 45 dB SPL, a threshold reported for mature crickets by Hedwig and Poulet (2005), before increasing to 55 dB SPL in very old animals. A reduction of threshold with age is also reported by Sergejeva and Popov (1994) for phonotaxis in flying *G. bimaculatus* of Central Asian origin, in which the threshold of 65 dB SPL at day 1 of adulthood decreased to 35 dB in mature females 11–20 days old. In *A. domesticus*, the phonotactic threshold decreased from 95 to 55 dB SPL over the first 4 days of adulthood (Walikonis et al. 1991). In young and mature female *G. campestris*, high sound intensities had a negative effect as phonotaxis decreased above 75 dB SPL, consistent with previous observations (Hedwig and Poulet, 2005; Schmitz, 1985). It is possible that high sound intensities indicate the close proximity of the signal source, and hence the need to reduce walking activity. The phonotaxis of the oldest adults to the best sound intensities of 75–80 dB SPL was only 66.7% as strong as the maximum response seen in young and mature crickets. This was equivalent to the response to 65 dB SPL songs shown by these younger crickets.

Measurements by Popov et al. (1994) of the mechanical properties of the tympanic membranes and acoustic trachea of *G. bimaculatus* indicate that the tympanum was functional within 1 day of becoming adult, but was somewhat less sensitive, particularly in the 5 kHz range of calling song. More challenging for phonotaxis was the finding of quite strong asymmetries in left and right tympanic performance in very young adult crickets and that sound propagation from the contralateral acoustic trachea was attenuated. Tympanic membrane sensitivity and sound propagation rapidly improved over the first 5 days of adulthood.

### Effect of carrier frequency

For female phonotaxis of *G. bimaculatus*, Popov and Shuvalov (1977) report the lowest threshold at 5 kHz. Threshold for phonotaxis showed a sharp increase towards lower and higher carrier frequencies, and frequencies higher than 10 kHz were not effective in eliciting positive phonotaxis and instead triggered negative responses. A corresponding frequency tuning curve for phonotactic responses in mature females is obtained if sound stimuli are presented at 75 dB SPL (Hedwig, 2016). In these previous reports and in our experiments, the range of strongest phonotaxis agrees with the carrier frequency of the natural calling song of *G. bimaculatus*, which is centred at 4.7 kHz and in the range of 4.3 to 5.2 kHz (Ferreira and Ferguson, 2002; Kostarakos et al., 2008, 2009; Verburgt et al., 2011). Throughout adulthood, the best phonotactic response occurred to test songs at 4.5 kHz, but 4–7 kHz songs reliably induced some phonotaxis. Within these boundaries there was evidence of a shift in preference with age: the relative attractiveness of 4 kHz song compared with 4.5 kHz song progressively decreased as crickets aged and, conversely, that of 5 and 5.5 kHz song increased in older crickets. This did not result in an overall expansion of the attractive frequency range as crickets aged; the 50% response range was widest in young and mature age crickets. Our data tentatively indicate that the most preferred frequency increases by approximately 250 Hz. This change in tuning with age is opposite to the change of the calling song carrier frequency in old males, which decreases by approximately 80 Hz (Jacot et al., 2007), with no effect on female preferences (Verburgt et al., 2011).

Age had a deleterious effect on overall performance to even the best calling song frequency; the phonotaxis of the oldest females was only 70% as strong as that of young and mature crickets in their prime, with responses to other frequencies similarly scaled down. The best responses of the oldest crickets were of a similar magnitude to the responses of younger crickets to less favourable 4 and 5.25 kHz songs.

### Effect of song pattern

The tuning of *G. bimaculatus* to songs with a systematic variations of pulse periods shows a broad best response in the range of 34 to 42 ms PP (Doherty, 1985b; Hennig, 2009; Schneider and Hennig, 2012; Hedwig, 2006), and is similar in the sister species *G. campestris* (Thorson et al., 1982). Although testing was done under different conditions, the tuning curves reveal that the response to PPs shorter than 30 ms generally decline more rapidly and 20 ms is hardly attractive, while the response to PPs higher than 42 ms decline more gradually towards a PP of 58 ms, which is not attractive. Within this range our data indicate subtle changes in song-pattern preference. The youngest crickets showed a nearly 40% difference in preference for 34 ms PP songs over 42 ms PP, but this relative preference decreased progressively, and in the old and very old age groups, there was no difference. The particular preference of the very young crickets has to be offset against their much lower level of activity overall – their peak response at 34 ms PP was only 35% as strong as the response of the young and mature animals to the same stimulus and only 40% as strong as the response of young crickets to the 42 ms PP stimulus. In young and mature crickets, which show the strongest phonotaxis overall, the relative preference for 34 ms over 42 ms PP was only 8–10%. There was evidence of a shift in preferred PP with age without a pronounced broadening of the breadth of patterns responded to. The relative attractiveness of 26 ms PP song decreased and the relative attractiveness of 42 and 50 ms PP songs increased in older animals (Fig. 3E). The overall width of the 50% response profile was similar in all age groups, but marginally wider in the young age group.

As with the other song parameters tested, the very old females showed on average only 68% as strong an absolute response to the 34 ms PP stimulus as young adults, and responses to other PP stimuli showed broadly similar magnitudes of reduced phonotaxis. The maximum responses of the very old crickets were broadly similar to those of young crickets to 26 and 48 ms PP.

The songs of male *G. bimaculatus* change as they age, the carrier frequency decreases by approximately 10% and pulse duration decreases by about one-third (Zhemchuzhnikov and Knyazev, 2015). Females reliably prefer the calls of younger males over those of older ones, regardless of their own age (Verburgt et al., 2011), provided the songs are of equal amplitude. Increasing the amplitude of older male songs above those of younger male songs in choice tests can overcome this innate preference for the song characteristics of younger males, and it seems that females may be trading off the distance they need to travel against male quality, because the louder sound source will tend to be nearer (Zhemchuzhnikov et al., 2017), akin to phonotactic responses to patterns of different quality and intensity in two-choice experiments (Gabel et al., 2015).

### Female choosiness

Substantial research has focussed on the effect of age on phonotaxis in different cricket species, but there has been no consensus on the degree of support for the female choosiness hypothesis (Tanner et al., 2019). According to life history theory, reproductive value is highest when females are able to take the time to select high-quality males, but that selectiveness declines with age, as the risk of dying without having mated increases.

In crickets, the choosiness hypothesis has been supported by studies, which however may require careful scrutiny. Prosser et al. (1997) tested *G. integer* females walking on a trackball system. Responses to different song patterns did not differ with age in single-choice experiments, and only in multiple-song-choice experiments were young females more selective than older ones. The authors emphasised that their data came with ‘a considerable amount of variation’ even when only responsive females were considered. If the total distance crickets will walk decreases with age, as our data indicate, then it will become more difficult to statistically discriminate between performances to different stimuli.

Gray (1999) reported that young (10 days old) female *A. domesticus* were more selective and spent more time choosing an attractive signal than older (20–21 days old) crickets. In two-choice experiments, females walked in a narrow trough with opposing speakers simultaneously presenting an attractive and a non-attractive song: females had to make a forced choice, while their peripheral auditory system has a limited ability to discriminate sound coming from the front and rear (Boyd and Lewis, 1983), which may be a confounding factor. Based on single-choice experiments, Walikonis et al. (1991) reported that the range of attractive PPs became broader with age in *A. domesticus*. However, phonotactic data are presented solely as relative measures of a female's walking response in a test. It is difficult to interpret such relative measures without considering the absolute measurements of responses. Females with a very low overall track length will score a high orientation component if they showed some deviation towards the sound source, and will be weighted the same as females that consistently walked towards the signal. Stout et al. (2010) analysed phonotaxis in four cricket species, scored as yes/no events based on rather broad criteria. Their data indicate that with age, *A. domesticus*, *G. veletis* and *G. pennsylvanicus* females may become unselective while in *G. bimaculatus* young (5–12 days old) and old (12–24 days old) females showed no difference in phonotactic selectiveness. In all four species, apparently unselective young females occurred, and the data do not reveal whether the unselective old females were also unselective when young. Therefore, substantial differences between *A. domesticus* and *G. bimaculatus* cannot be excluded, but as Stout et al. (2010) point out, quantitative measurements of phonotaxis would have provided a more informative dataset.

Other behavioural studies do not support the choosiness hypothesis: e.g. Kuriwada and Kasuya (2006) on *Meliomorpha japonica* with a two-choice paradigm, and Pacheco et al. (2013) on *G. assimilis* using a no-choice trackball-based paradigm across age groups spread over 28 days. Our data are in broad agreement with Pacheco et al. (2013), who found that on days 1–7 there was little response or reliable orientation, and that the greatest movement towards the signal occurred on days 10–13, but older females, while reliably orientating towards the signal, walked much more slowly.

We did not see the broadening of song parameters that elicit phonotaxis with increasing age that the female choosiness hypothesis predicts. The proportion of individuals that did not perform phonotaxis to an attractive song does however support one aspect of life history theory. Very young animals readily engaged in phonotaxis, but the frequency of non-phonotactic days increased in middle-aged crickets. As crickets became very old however, the incidences of non-response decreased, consistent with the hypothesis that not taking a mate-finding opportunity when they have little life expectancy left increases the risk of having no offspring. In a predation context, older *Teleogryllus oceanicus* females took greater risks and walked further exploring their environment, as they may have less to loose in terms of life expectancy (Moschilla et al., 2018).

Despite their short lifespans, insects display signs of aging (López-Otín et al., 2013). In cockroaches, age causes degeneration of the nervous and musculoskeletal systems, leading to reduced activity and difficulties in complex locomotor tasks (Ridgel and Ritzmann, 2005). Such processes may contribute to the gradual decrease of phonotaxis in aging crickets. The 30–33% decreases in responsiveness shown by the oldest crickets compared with young crickets across all song parameters suggest an overall decrease in motor capacity, rather than specific adjustments to different song elements.

Cricket phonotaxis has been used as a proxy to explore selectiveness related to age, but it is only a first step as the final mating decision depends on nearfield communication involving courtship song (Balakrishnan and Pollack, 1996) and further sensory cues (Hissmann, 1990; Adamo and Hoy, 1994; Tregenza and Wedell, 1997; Simmons et al., 2013). Attraction to calling song may not be a sufficient predictor of final mate choice. Based on the range of phonotactic responses we observed, female *G. bimaculatus* undergo only small changes in song selectivity throughout their adult lifetime, but there is a profound difference in their overall responsiveness as they age.
